# A Glucose Sensing System Based on Transmission Measurements at Millimetre Waves using Micro strip Patch Antennas

**DOI:** 10.1038/s41598-017-06926-1

**Published:** 2017-07-31

**Authors:** Shimul Saha, Helena Cano-Garcia, Ioannis Sotiriou, Oliver Lipscombe, Ioannis Gouzouasis, Maria Koutsoupidou, George Palikaras, Richard Mackenzie, Thomas Reeve, Panagiotis Kosmas, Efthymios Kallos

**Affiliations:** 1 MediWise| Medical Wireless Sensing Ltd, Queen Mary Bio Enterprises Innovation Centre, (QMB), 42 New Road, London, E1 2AX UK; 2 School of Natural and Mathematical Sciences, Kings College London, London, WC2R 2LS UK; 3 Institute of Life Sciences, University of Roehampton, London, SW15 5PJ UK

## Abstract

We present a sensing system operating at millimetre (mm) waves in transmission mode that can measure glucose level changes based on the complex permittivity changes across the signal path. The permittivity of a sample can change significantly as the concentration of one of its substances varies: for example, blood permittivity depends on the blood glucose levels. The proposed sensing system uses two facing microstrip patch antennas operating at 60 GHz, which are placed across interrogated samples. The measured transmission coefficient depends on the permittivity change along the signal path, which can be correlated to the change in concentration of a substance. Along with theoretical estimations, we experimentally demonstrate the sensing performance of the system using controlled laboratory samples, such as water-based glucose-loaded liquid samples. We also present results of successful glucose spike detection in humans during an *in-vivo* Intravenous Glucose Tolerance Test (IVGTT). The system could eventually be developed into a non-invasive glucose monitor for continuous monitoring of glucose levels for people living with diabetes, as it can detect as small as 1.33 mmol/l (0.025 wt%) glucose concentrations in the controlled water-based samples satisfactorily, which is well below the typical human glucose levels of 4 mmol/l.

## Introduction

The millimetre (mm) wave band of the electromagnetic (EM) spectrum offers valuable spectroscopy methods for material characterization^1–3^. The characterization can be performed in free space and does not require very precise alignment, as is the case for optical frequencies. Moreover, the mm frequency range is ideal for characterization of composites with scale of homogeneity in the order of millimetres^1^.

Quasi-optical setups have been used recently for dielectric measurements to detect the water contents of rocks and oils^1^. The measurement system proposed in ref. 1 is based on waveguides and requires additional components such as lens, filters, etc., which lead to additional complexity. Coplanar waveguide devices have also been used for dielectric spectroscopy of biological samples at 40 Hz–26.5 GHz^2^ by placing a liquid sample on the waveguide structure, thereby providing an accurate but non-invasive technique. Moreover, two-beam polarization interferometry spectroscopy has been used up to 300 GHz to characterize the dielectric constants of low loss polymer samples, where a complex setup including polarizer, mirror, and beam splitter has been used to perform the measurements. In addition to material characterization, mm-wave spectroscopy in reflection mode has also been used to detect sugar or glucose levels in water or blood, in the frequency spectrum of 33–95 GHz^4^.

To date, most commercially available glucose measurement methods are invasive by relying on drawing blood, and thus patients do not test as often as required for the proper management of diabetes. Non-invasive continuous glucose monitoring devices have been sought after for more than four decades^5^. With regard to glucose sensing in particular, various methods utilizing optical or lower frequency EM waves have been examined in the past^6–9^. For example, a dielectric resonator operating at 1.68 GHz has been developed to detect glucose changes based in its resonant frequency changes^9^, but with a detection resolution (5 mg/ml) that is much lower than that required for humans. The mm-wave band is particularly attractive for this application^7, 8, 10^, as the wavelength is short enough for a relatively compact antenna sensor, and the penetration depth is large enough for interrogation of thin human tissue regions with sufficient blood concentration. Mm-wave spectroscopy in reflection mode has been used for non-invasive glucose sensing through human skin^7^. A challenge in reflection-based detection is that the signal barely reaches the blood area and this minimal interaction may yield information that is not sufficient to detect small changes in glucose levels. To take advantage of transmission data, a sensor operating at K band (27–40 GHz) has been developed to detect glucose level in blood non-invasively^8^. The device used waveguide fixtures and was tested in rats on tissue of 0.5 mm thickness, which is much thinner than typical human tissue (2–3 mm).

To assess the possibility of detecting glucose by mm-wave technology, a measurement system using WR-15 V-band waveguides operating at 50–75 GHz has been demonstrated by the authors^10^. The system used transmission data to detect glucose concentration changes of clinical relevance in either pure water or saline solutions. The samples were contained in an acrylic tank which was sandwiched between two open waveguides. However, the dimensions of this measurement system were large, and the experimental setup required sophisticated alignment to accurately measure the transmission and reflection coefficients. Therefore, the bulky waveguides must be replaced by compact planar antennas with mm dimensions in order to develop a compact and miniature sensor which is comfortable and easy to use by patients.

**Figure 1:**
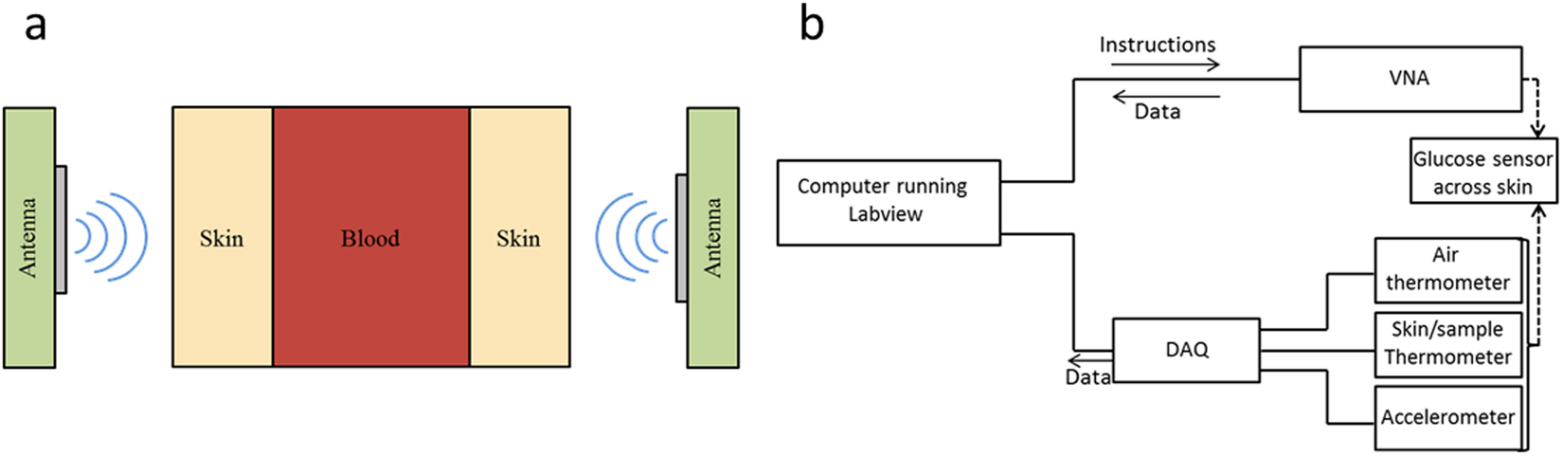
The sensor design and principles. (**a**) The mm-wave glucose sensor concept set-up with two patch antennas. The skin/blood/skin patient area or a tank is placed in between the two antennas. (**b**) A schematic of the sensor system with data and instruction flow controlled by Labview.

This paper describes a sensing system towards the realization of a handheld device, which can detect complex permittivity variations and correlate them to changes in the concentration of a specific substance, such as glucose concentration in water or blood. A pair of patch antennas tuned to operate around 60 GHz has been designed and fabricated to replace the earlier-used waveguide structures. These antennas are mounted on an experimental setup for measuring transmission across an acrylic tank filled with controlled water-based glucose solutions using micro-positioners and 3D-printed sample holders. The micro positioners can align the antenna against each other precisely to maximize the transmission across the tank. A schematic view of the proposed detection principle is shown in Fig. 1(a), where two identical antennas are placed across the sample of interest, e.g. human tissue. The antenna sensors are mounted inside a U-shaped casing and the area under measurements (AUM) is placed in-between the extended arms of the casing^11^. Other than the RF components, the sensor as shown in Fig. 1(b) also includes additional environmental sensors to monitor the impact of external factors such as motion and temperature. Precise measurements and data interpretation are achieved by an algorithm which integrates the data from the antennas and the complimentary sensors and translates this information into detection of glucose level variations.

The paper consists of three parts. First, a theoretical estimation of the system’s optimal frequency to maximize the sensitivity of detecting glucose levels within the uncertainty limits of the system’s hardware in presented. We also argue why the mm-wave range is beneficial for measuring concentration changes across mm-thick samples. The sensitivity of the sensor depends on both received power and the noise floor of the instrument, i.e. the signal to noise ratio (SNR). Second, we experimentally evaluate the system’s sensing capability using different types of controlled samples, such as attenuators, tanks with water-based glucose-loaded samples, and human tissues. To assess the glucose monitoring performance in the glucose loaded samples, the transmission across the tank filled with various concentrations of glucose solution has been measured and compared with pure water. The results show a satisfactory correlation between transmission coefficients and glucose concentration, and can detect as low as 0.025 wt% of glucose in water. Finally, we present *in-vivo* glucose sensing measurements in healthy humans undergoing a standard Intravenous Glucose Tolerance Test (IVGTT). The *in-vivo* results show that despite the signal uncertainty introduced by the human tissue, glucose spikes can be adequately detected with the present sensing system.

## Results and Discussions

### Estimated system sensitivity, uncertainty and optimal sensing frequency

Before evaluating our proposed system with experimental measurements, we provide estimates for two of its key aspects, its sensitivity and measurement uncertainty, which can assist in the choice of the system’s optimal sensing frequency. The system’s sensitivity is equivalent to the expected recorded transmission signal difference for a given change in the glucose concentration, measured in dB per wt% or dB/per (mmol/l). Since we utilize glucose dissolved in water-based samples, its concentration can be measured as a percent (wt%) w/w in water, or simply through the weight of glucose (in mmol) per 1 l of water (mmol/l). For example, 1 wt% concentration is equivalent to 55.6 mmol/l, given that glucose has a molecular weight of 180.2 g/mol. The measurement uncertainty of the developed system can be defined as the maximum measurement error in the recorded signal (in dB) which is allowed for sensing a given value of glucose concentration, given that the ISO: 15197:2013 standard allows a ± 0.86 mmol/l maximum error in measuring low blood glucose levels^12^.

#### Sensitivity

An order of magnitude estimate for the system sensitivity can be calculated as follows. For a change Δ*g* in glucose concentration (in mmol/l or wt%), let Δ*S*_*21*_ be the measured amplitude variation of the transmitted signal *S*_*21*_ at frequency *f*, which propagates through a sample of thickness *d*. The dominating factor affecting the transmitted signal is the change of the imaginary part of the relative permittivity *ε*_*g*_ of dissolved glucose. The permittivity *ε*_*g*_ typically follows a Debye distribution^13^, and the imaginary part of *ε*_*g*_ (*ε*_*g*_″) decreases with increasing glucose concentration, leading to an increase Δ*S*_*21*_ in *S*_*21*_. The decrease in *ε*_*g*_″ with increasing *g* is well known and has been measured by the authors^10^ and by other researchers^14, 15^. For example, a Δ*g* = 1 wt% for *d* = 1 mm at 60 GHz will decrease *ε*_*g*_″ by ~0.23 units^10^.

The power transmission through a slab of glucose solution with complex permittivity *ε*_*g*_ (*g*, *f*) is dominated by the exponential loss factor as follows:

1 $${S}_{21}(dB)=20\,\mathrm{log}|{e}^{-j{k}_{0}d\sqrt{{\varepsilon }_{g}(g,f)}}|,$$

where *k*_*0*_ is the free space wavenumber at frequency *f*. This equation is valid under the following assumptions: (i) plane wave incidence; (ii) edge effects from the transverse slab boundaries are neglected; (iii) reflections from the air-slab interfaces are also neglected, and so is the presence of additional layers on either side of the glucose slab, such as plastic holding tank layers. In fact, when we calculated the S_21_ value with these additional layers present using more complex transmission formulas^16^, the difference in the sensitivity value was no more than 10% from the value presented below.

The complex permittivity of the glucose solutions as a function of both concentration (up to 5 wt%) and frequency (in the 40–67 GHz range) was measured in earlier work by the authors^10^ and fitted using a 1^st^ order Debye model, ${\varepsilon }_{g}(\omega )={\varepsilon }_{\infty }+\frac{{\varepsilon }_{s}-{\varepsilon }_{\infty }}{1+i\omega \tau }$. For example, the Debye parameters were calculated to be, *ε*_∞_ = 5.6184, *ε*_*s*_ = 78.5250 and *τ* = 8.8601 ps for g = 1 wt%. The estimated sensitivity of the system for given values of d and g is calculated numerically from the above equation as

2 $$\begin{array}{rcl}\frac{{\partial }^{2}{S}_{21}}{\partial g\partial d}(g\le 1\,wt \% ;\,d\le 5\,mm;f=60\,GHz) & \approx  & 0.26\frac{dB}{mm\cdot 1 \% }\frac{dB}{mm\cdot 1\,wt \% }\\  & = & 0.0047\frac{dB}{mm\cdot (mmol/l)}.\end{array}$$

This sensitivity value is mostly dependent on the Debye model of the glucose permittivity, and it is very weakly dependent on the glucose concentrations values and the slab thickness *d*. It also depends strongly on frequency: for a fixed slab thickness *d*, the factor *k*_*0*_ *d* in the exponential of the equation above increases. Another way to view this is that the slab becomes electrically longer as the wavelength decreases, causing higher losses and thus larger differences in *S*_*21*_.

The values presented in the above equation are valid for relatively small values of *g* (up to 1%) and *d* (up to 5 mm). As an example, for a tissue thickness of *d* = 2.5 mm, which is typical for human body sections such as the earlobe and the tissue between the fingers, and if a glucose change of Δ*g* = 0.86 mmol/l is to be detected^12^, Eq. (2) above indicates that a signal of 0.010 dB should be reliably detected.

#### Uncertainty

**Figure 2:**
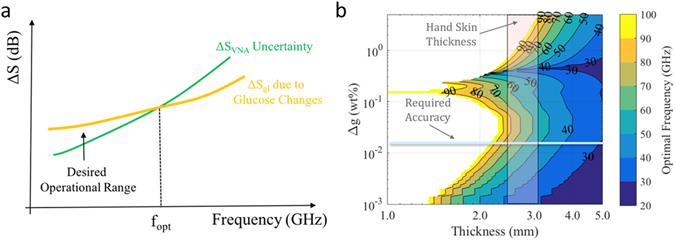
Sensitivity and optimal frequency of operation. (**a**) Optimal frequency operating range considering difference in transmission coefficient and system uncertainty. (**b**) Optimal frequency of operation for different glucose concentrations and sample thicknesses.

To increase sensitivity, the difference in transmission data Δ*S*_*21*_ due to a glucose concentration change Δ*g* should be as high as possible. For a given sample thickness *d*, Eq. (1) indicates that Δ*S*_*21*_ increases with frequency. The difference in the imaginary permittivity values for two different glucose concentrations will result in a higher Δ*S*_*21*_ for electrically longer samples, as illustrated in Fig. 2(a) (yellow line). The operational frequency, however, cannot be arbitrarily increased as electrically longer values will lead to smaller values for *S*_*21*_, which eventually will run into the noise floor of the sensing system and will not be accurately measured. It is well known that for Vector Network Analyzers (VNAs) the measurement uncertainty improves with increased Signal to Noise Ratio (SNR)^17^. For example, for the Keysight E8361 VNA used in our system, the measured dependence between uncertainty U and SNR at the receiving port for 10 Hz IF bandwidth is^18^

3 $$U[dB]\approx 6.04{e}^{-0.12\ast SNR[dB]}$$

From the above equation, a required uncertainty of 0.01 dB (necessary to detect a Δ*g* = 0.86 mmol/l, as argued in the previous section) requires an SNR of 55 dB or better.

A higher SNR will lead to a smaller value of Δ*S*_*VNA*_ that can be accurately measured. At the same time, operating at higher frequencies leads to lower SNR values, as the signal attenuates more for electrically longer samples. The end result is that the uncertainty of the sensing instrument, i.e. the smallest values of Δ*S* that can be reliably measured, or Δ*S*_*VNA*_, will also increase with frequency. This is shown also in Fig. 2(a) (green line).

#### Choosing the optimal sensing frequency

To achieve accurate glucose sensing, the sensing system should be designed in a way that its highest sensitivity (the Δ*S* value achieved at *f*_*op*t_) is above the Δ*S*_*21*_ due to the lowest desired glucose change Δ*g* that must be detected (for example, 0.86 mmol/l^12^). To provide a graphical interpretation of this process, we have calculated the optimal frequency *f*_*opt*_ for different values of sample thickness *d* and glucose concentration *g* in %, assuming that the glucose is dissolved in water. We investigated the frequency range of 20–100 GHz over which the Debye models for the permittivity of the different glucose concentrations are relatively well known through measurements^10^ and the results are shown in 2(b).

The results in 2(b) consider that the power input onto the sample is 0 dBm, which is a typical output achieved by many modern VNAs. This value lies well below the IEEE safety radiation exposure limit for these frequencies, which is 20 dBm^19^. Moreover, the achievable noise floor value of our sensing system is set at −114 dBm. This value was measured in our experiments and is also in line with product specifications provided by the VNA manufacturer (Keysight). The transmission differences through the different samples were calculated using an analytical model of a transfer matrix method for transmission through multiple slabs^15^; however, the simplified Eq. (2) yields very similar results.

Figure 2(b) confirms that *f*_*opt*_ decreases with increasing sample thickness, in order to maintain the electrical length of the sample. For the regions shown in white, the predicted f_opt_ is above 100 GHz, for which accurate Debye model data for the glucose permittivity are not available. Also, *f*_*opt*_ mostly increases with increasing Δ*g*, with the exception of a region between Δ*g* between 0.1 and 0.2. This behavior can be traced back to the Debye models obtained for particular glucose concentrations. Figure 2(b) can be used to provide an optimal frequency range for sensing glucose levels down to a minimum level of glucose concentration; for example, for a desired sensing accuracy of glucose change Δ*g* = 0.86 mmol/l or higher, and a typical range of tissue thicknesses for hands 2.5–4.0 mm, the highlighted region in Fig. 2(b) confirms that the optimal sensing frequency lies in the middle of the mm-wave spectrum, in the range of 40–80 GHz.

To summarize, Fig. 2(b) quantifies the theoretical analysis of this section which has argued that a frequency around 60 GHz is desirable for glucose detection using EM transmission data. For increasingly higher frequencies, the losses are prohibitively high for the SNR to exceed the noise level of a sensing instrument such as a VNA, in order to maintain good measurement uncertainty. On the other hand, much lower frequencies result in changes in the glucose sample permittivity which are below the system sensitivity and cannot produce a measurable transmitted signal Δ*S*_*21*_.

### Glucose sensing results in controlled environment

In this section, we examine the glucose sensing capabilities of the system. First, we investigate the limits in measurement uncertainty. Second, we study the effects of environment temperature and motion on the received S_21_ signals. Finally, we present glucose sensing data in controlled water-based glucose-loaded samples of varying concentration to estimate the low-concentration capabilities of the system.

#### Assesment of the measurement uncertainty of the system hardware

Considering for the moment that variations due to external factors are minimal, the inherent noise component of the signal affects the measurement as the noise adds either in-phase or out-of-phase to the measured signal. This error is independent of the quality of the instrument, as long as its noise floor is lower than the signal’s power level. If the received signal shows no distortion products and the VNA’s noise floor is below the signal with a sufficient dynamic range, the uncertainty depends solely on the signal to noise ratio (SNR) of the received signal.

**Figure 3:**
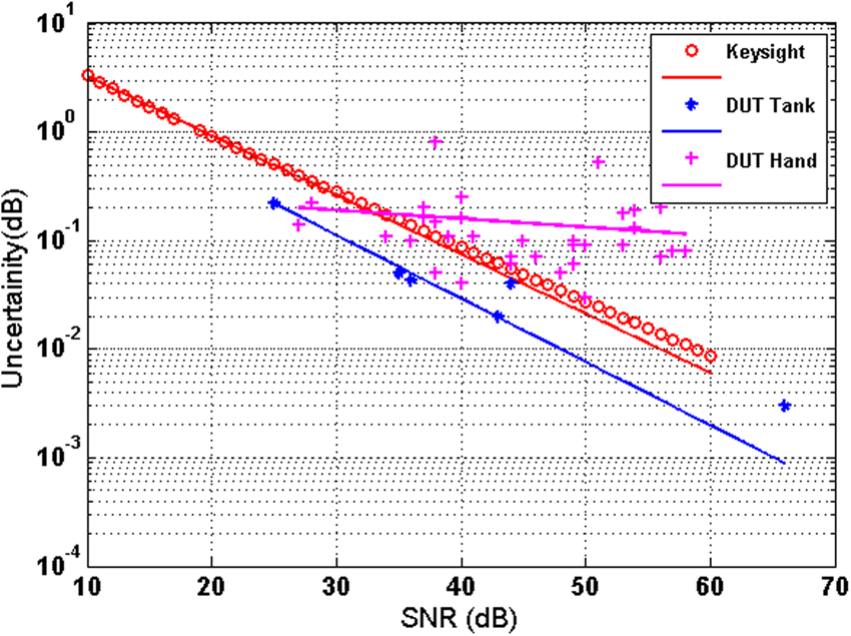
Measurement uncertainty as a function of SNR: Different devices under test using the Keysight E8361A VNA. For the case of glucose-loaded water-based samples and hand, the SNR is calculated by dividing the received signal at port by the noise floor provided by Keysight from characterization data sheet.

In order to assess the received signal uncertainty as a function of the SNR, we compare three distinct measured quantities of uncertainty (the standard deviation or error) in Fig. 3: (i) The manufacturer-provided uncertainty of the VNA^18, 20^; (ii) The measured uncertainty of transmission measurements through a 1 mm-thick glucose-loaded water-based sample; iii) The measured uncertainty of transmission through human hand tissues of varying thickness (and hence varying SNR).

The results in Fig. 3 show that good sensitivity levels, close to the ones can be provided by the VNA instrument, are maintained when non-human samples are involved. The uncertainty curve deteriorates when measuring transmission in human tissue due to the hand motion during measurement. Clearly, the uncertainty can be improved with increasing SNR, and thus the resolution of detection be improved. For a fixed noise floor, the SNR can be improved by increasing the received signal and for a certain instrument the SNR can be enhanced by amplifying the signal after the source or before the receiver as the signal is transmitted through the sample.

Assuming a typical (for the Keysight VNA utilized in this work) SNR of 60 dB, the uncertainty of the system as shown in Fig. 3 is calculated to be 0.006 dB for non-human tissues. It can be seen that the smallest glucose levels that the sensing system hardware can detect is as small as 0.024 wt/wt in the water, or 1.33 mmol/l of concentration in blood (ΔS_21_ = 0.25 dB for 1% glucose solution for a 3-1-3 tank, as shown later in II.C). Thus, the glucose sensing system hardware is capable of resolving clinically meaningful blood glucose concentrations. However, the external errors, the environmental variables, the human tissue motion, and the true characteristics of the signal (power, noise level, spurs, etc.) will affect the sensitivity of the measurement. The sensitivity can be improved further using the full potential of the amplifier (∼10 dB gain), which would reduce the detection limit to one third (~0.4 mmol/l).

#### Assesing the impact of environmental factors

**Figure 4:**
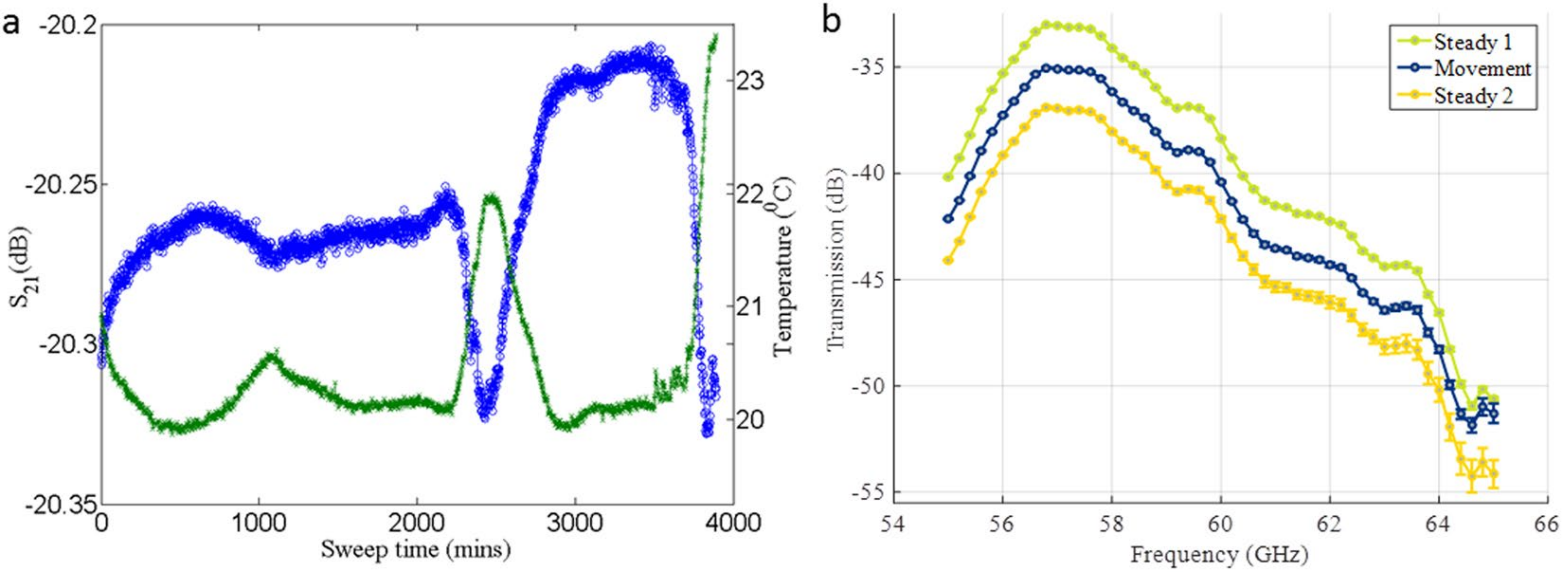
The effect of environmental factor on measured data. (**a**) The variation in S_21_ and temperature for a 20 dB attenuator recorded over a two days period. (**b**) The effects of vibration (movement) on the S_21_ through a 3-1-3 tank using phase-stable cables. The curves have been offset from the original values in the S_21_ axes to better illustrate the errors introduced. Steady state 1 and 2 means measurement before and after vibration.

We have also measured the effect of environmental factors such as temperature and vibration on the transmission coefficient S_21_. To assess the temperature effects, a 20 dB attenuator was connected to the VNA and both ambient temperature and S_21_ were measured continuously over a period of two days. The recorded data are shown in Fig. 4(a). It can be seen that a negative temperature coefficient has been observed in S_21_, i.e. when the temperature increases S_21_ decreases and vice versa. This allows to correct the S_21_ for a certain sample to measure actual variations due to permittivity changes. We have also measured the variation in S_21_ with vibration on the cables, as shown in Fig. 4(b). The results show that there is no systematic drift with vibration other than the higher standard deviation (up to 2 dB) as vibration strengthens, which is more prominent at higher frequencies. The effect is more prominent in a non-phase stable cable (not shown). The current setup does not use this data to auto-correct the measurement results; however our future system will integrate data from these sensors controlled by a LabVIEW interface to correct the RF measurement data and predict the dielectric constant or glucose concentration in real time.

#### Correlation of transmission data to glucose concentrations of water-based glucose-loaded samples

**Figure 5:**
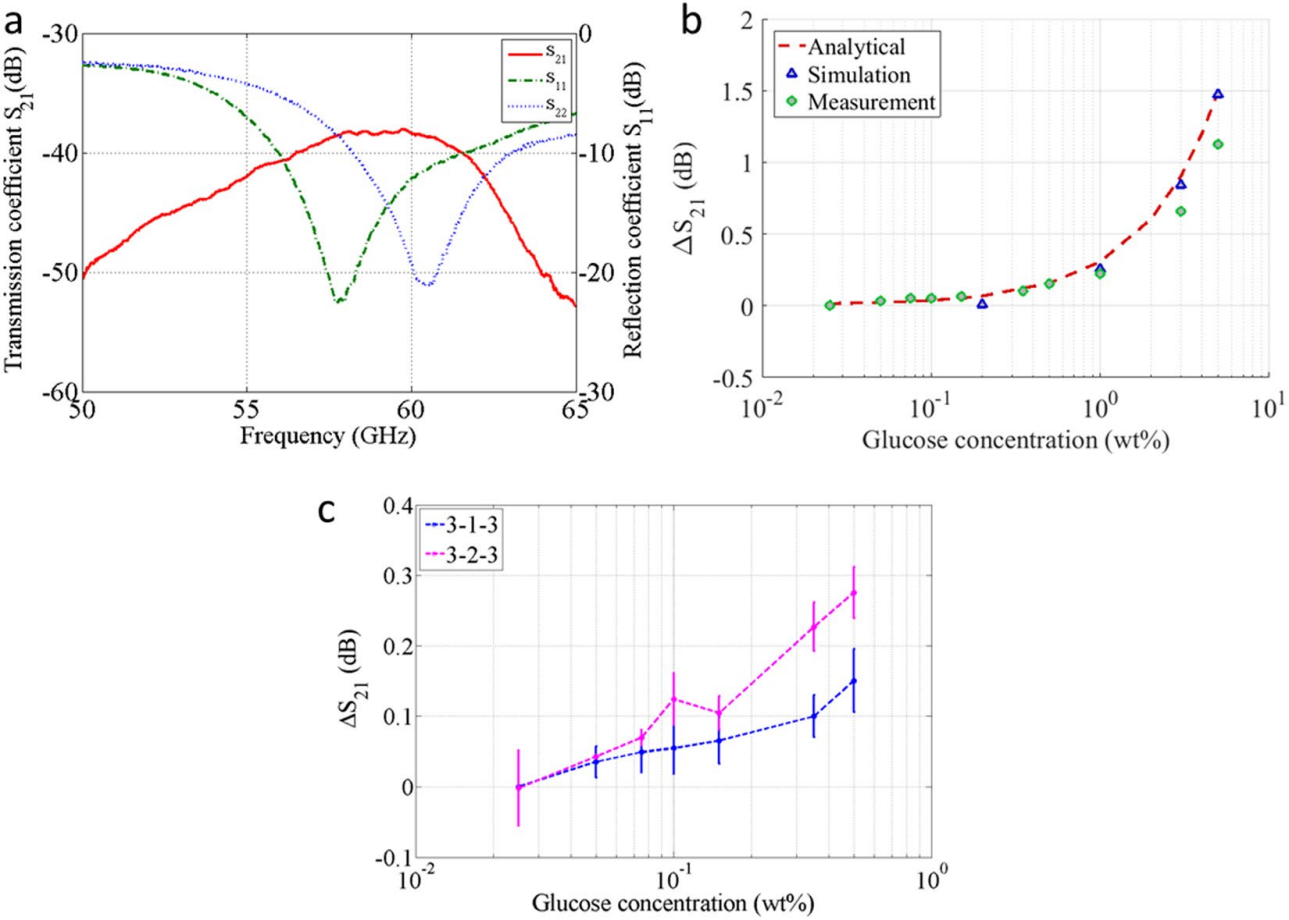
The correlation between transmission coeffcient and glucose concentration. (**a**) Reflection and transmission coefficient vs. frequency for a 3-1-3 tank filled with pure water (**b**) ΔS_21_ for various glucose concentration from measurement, simulation and analytical calculation @ maximum trasnmission coefficient. (**c**) ΔS_21_ for 0–0.5% (smaller) glucose concentration from transmisison measurement for 3-1-3 and 3-2-3 tank @ maximum trasnmission coefficient. The plot has been shifted to zero for 0.025% since pure water (0%) is not seen in log scale. X axis has been plotted in log scale for both b & c to distinguish the lower concentrations. The error bar are standard deviation from three sets of measurements.

In this section we present measurements of the transmission coefficient across an acrylic tank filled with a liquid glucose solution. The acrylic-spacer-acrylic tank has thicknesses of 3.0, 1.0, and 3.0 mm respectively (“3-1-3” tank), and is kept fixed during the measurements, as shown in the methods. A two-port calibration is performed using an electronic calibration kit before each set of measurements. Then, *S*_*21*_ is optimized to provide maximum transmission by moving the antennas symmetrically against the tank filled with deionized water. As shown in Fig. 5(a), maximum transmission was obtained when both antennas had minimum return loss. Having optimized *S*_*21*_ with water, the tank was filled with glucose solutions of various concentrations and the *S* parameters were recorded for each solution. The transmission coefficient for water was subtracted from that of various concentrations of glucose to get the absolute difference for each concentration.

Figure 5(b) shows the variation of Δ*S*_*21*_ for various concentrations of glucose at 58 GHz, where the transmission is maximum. The Δ*S*_*21*_ in dB varies linearly with the glucose concentration. We have compared the experimental results with results both from 3-D EM simulations for various concentrations of glucose, as well as analytical calculations assuming plane wave propagation^10^. For both these calculations, the dielectric constant for each glucose concentration was obtained from measurements using a dielectric assessment kit (DAK), DAK-1.2E probe from SPEAG that operates up to 67 GHz. It can be seen that the measured Δ*S*_*21*_ results agree well with both simulation and analytical data. In particular, the plots in Fig. 5(b) suggest that Δ*S*_*21*_ is equal to around ~ 0.25 dB for each 1 wt% of glucose concentration, which matches the analytical and simulation data closely.

To show the capability of the device to detect clinically meaningful glucose levels for humans, the lower concentrations solutions are measured in both 3-1-3 and 3-2-3 tank and shown in Fig. 5(c). The linear relationship resembles the trend shown at higher concentrations. The lower than expected Δ*S*_*21*_ for thicker sample is attributed to surface wave that bypass the water/tissue, contributing to the overall transmission coefficient. The standard deviations from three consecutive measurements are also shown in the plot and these can be attributed to human imperfections during refilling the water tank, sample temperature variations, and the VNA drift due to temperature and other environmental factors which could be eliminated. The results show that a concentration as small as 0.025 wt% produces a detectable change on ΔS_21_ compared to adjacent concentrations, and could be used to detect human level glucose concentration in optimum measurement conditions.

### *In-vivo* human clinical investigation and comparison with invasive methods

The sensing system was tested for real-time *in-vivo* blood glucose monitoring during human clinical investigation at the Department of Life Sciences, University of Roehampton, London, UK. Ten healthy male volunteers (n = 10) underwent an *in-vivo* Intravenous Glucose Tolerance Test (IVGTT). Blood glucose concentrations were continuously measured. Simultaneously to the sensing system attached to the volunteer’s right palm, blood samples were drawn from their left hand and measured with a lab analyser (Biosen C-line) at regular intervals, over a period of approximately 60 minutes. Each session started with 5 min baseline measurements, followed by the administration of an iv glucose load to the left arm over a 2-minutes-period, in order to create a physiological high blood glucose spike. The detailed protocol is described in Section D (Methods). The results presented good correlation between recorded transmission coefficient (S_21_) data and the reference data collected with the invasive method for two subjects (n = 10). Data collected from the other eight volunteers showed distortions in the received radio-frequency signal suggesting hand motion and gradual sliding of the holder during the session, possibly because of fatigue or stress.

**Figure 6:**
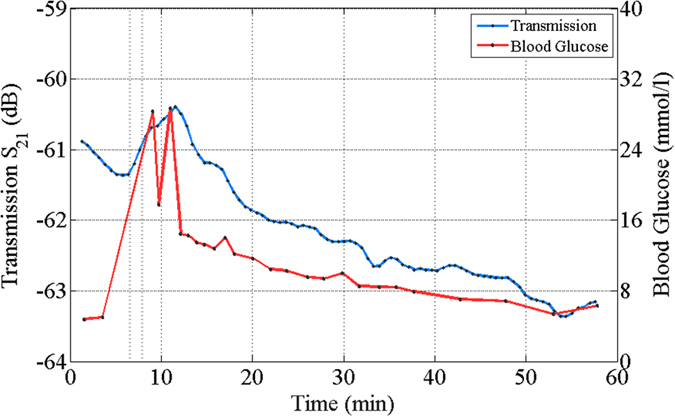
The *in-vivo* measurements results and comparison with invasive methods. The relation between measured transmission coefficient and glucose reading from a conventional glucose meter with time. There is a delay (~3 min) in the glucose spike between invasive method and our peripheral data. GlucoWise’s conversion to mmol/l matches well with the invasive methods. The trial was conducted at Roehampton University, London.

Figure 6 shows an example comparison between the continuous non-invasive glucose reading and the data collected invasively for a volunteer that produced good correlation. The variation in transmission coefficient (S_21_) with time follows the trend closely shown by invasive method, successfully detecting the glucose spike. The glucose concertation as measured by the reference method increases from 6 mmol/l to 30 mmol/l, a change of 24 mmol/l. Based on the theoretical calculations and the controlled sample sensing measurements presented in earlier sections, where a 0.00047 dB change per mm of tissue thickness for every mmol/l of glucose change is expected, we can make an order-of-magnitude estimate of the expected change in S_21_ due to the produced glucose spike. Using the 5 mm-thick value for the particular subject’s tissue thickness, the expected total change is ΔS_21_ = 0.0047 dB/(mm*mmol/l) * 5 mm * 24 mmol/l = 0.56 dB. In the investigation, a change of 0.8 dB was measured, which falls within the order-of-magnitude estimate. The difference is attributed to the possibly higher actual glucose concentration at the hand location that the sensing system was placed, and the more complex structure of the human tissue than the one assumed in earlier the calculations^21^.

The reference invasive method shows that the glucose spike occurred during the 9^th^ min. According to the S_21_ measured signal, the spike is observed during the 12^th^ min. The 3 min delay for the S_21_ spike is attributed to the time required for the blood to flow from hand to palm^22^. In addition, while for the invasive method the blood was collected from the vein, the sensing system was attached to and measured from peripheral region inducing further delay. The current device did not measure specificity of glucose over other substance in blood; however the device specificity of glucose over salt has been shown in an earlier piece of work by the authors^10^. Future work would include improving the repeatability, measure lower concentration of glucose *in-vivo* and specificity of glucose over various blood substances.

In conclusion, we have developed a miniature prototype for mm-wave dielectric permittivity measurements with focus on biomedical applications, which is herein studied for the detection of glucose concentrations in liquid samples and *in-vivo* human subjects. A set of 1.50 mm by 1.50 mm square patches have been designed and fabricated to be used as both transmitter and receiver element with a VNA. The prototype has been used to measure various concentrations of glucose solution contained in an acrylic tank. The measured Δ*S*_*21*_ relative to pure water changes linearly with glucose concentration, and the sensitivity per 1% substance match theoretical limits and are comparable to waveguide-based measured data. The device has performed *in-vivo* clinical investigation in human and successfully measured larger glucose level induced by intravenous glucose load. Based on these findings, the development of a complete miniature handheld device is under way, where the VNA will be replaced by a compact and integrated source and detector. In order to increase the sensitivity for thicker samples and hand tissue, absorber will be used around the antenna to block the surface wave bypassing the region of interest i.e. tissue or glucose solution. Environmental effects have also been integrated in the development of the sensing system, with the goal to cancel the impact of heat and motion/vibration in the measurements.

Beyond biomedical applications, the proposed sensing system can be used in applications where continuous monitoring of changes in the dielectric constant or conductivity of a liquid is required, for example allowing real-time monitoring of changes in the substance of a specific container without opening it. Our driving healthcare application is continuous monitoring of glucose levels in diabetic patients without the need of drawing blood, where we have targeted an accuracy of 0.025 wt% of glucose in blood.

## Methods

### System design

As mentioned in the Introduction, the main components of this mm-wave permittivity measurement system is a pair of 60 GHz antennas, which are powered and measured using a Keysight E8361A VNA. To monitor external effects which could lead to variations in the performance of the sensor especially in live biological measurements, environmental sensors were mounted within the antenna holder (see Fig. 1(b)). This set of sensors includes two thermometers (one to measure the sample or skin temperature, and one to monitor the ambient air temperature), and a solid-state three-axis accelerometer to detect movement. These sensors were monitored and controlled by National Instruments data acquisition hardware (DAQ) which in turn communicated to a computer running LabVIEW. An in-house LabVIEW program was developed so that the VNA S-parameter results can be viewed alongside the sample/skin and air temperature and sample/skin movement as a function of time. This feature can assist in removing external ambient effects, for example by allowing data to be removed at times when anomalous vibration is observed. In future work, the sensors will be used to correct or modify the data, thereby performing a real-time system calibration.

### Standalone Antenna Simulation, Fabrication and Characterization

**Figure 7:**
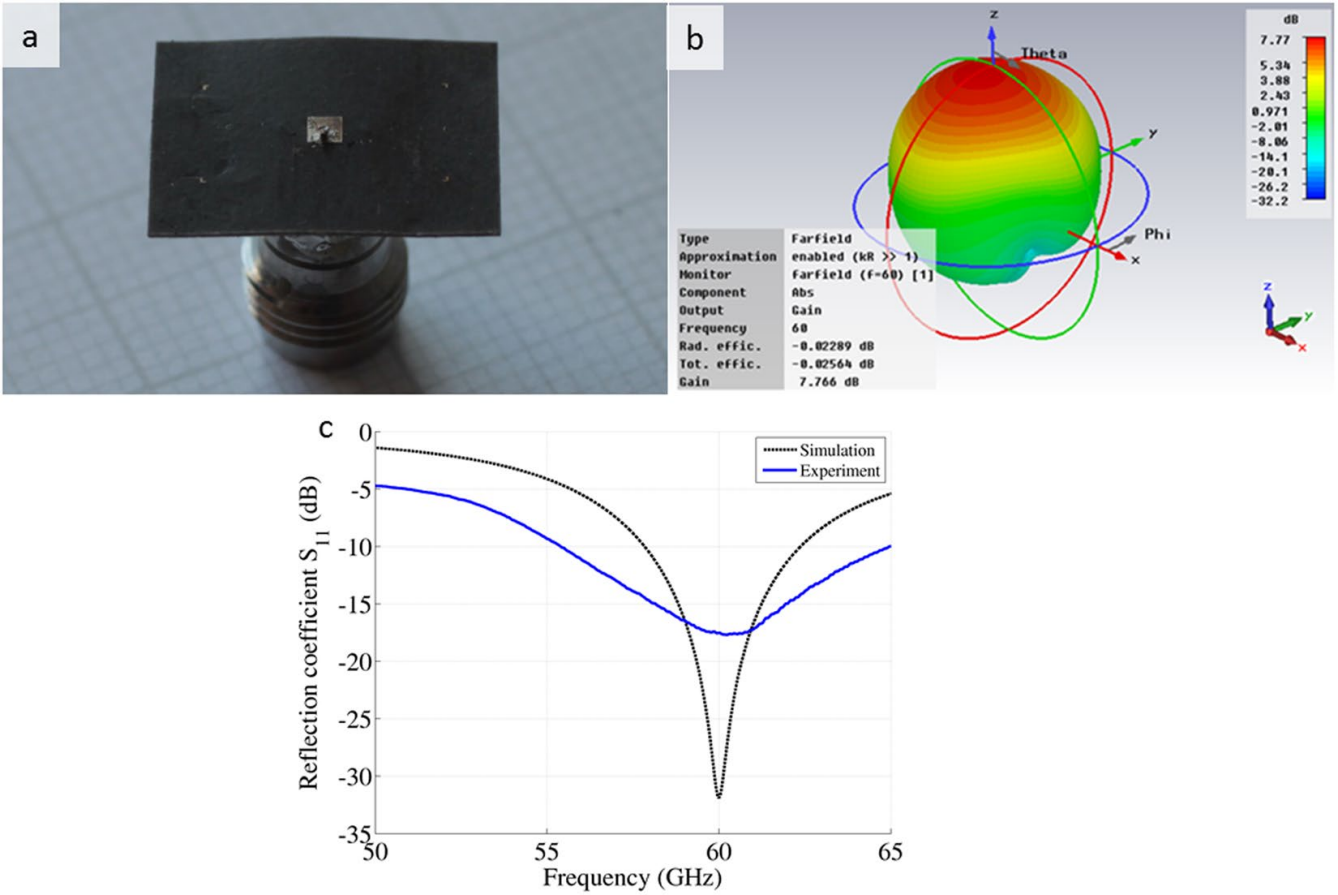
The antenna design, simulation and characterization. (**a**) Photo of the square patch, soldered from the back side. (**b**) Simulated 3D radiation pattern of the antenna gain at 60 GHz, which is the desired operating frequency of the antenna. (**c**) Free space simulated and measured return loss (*S*_*11*_) for the antenna of Fig. (**a**).

Rectangular microstrip patch antennas have been designed as the basic element for the sensor, with a Rogers 5880 substrate material of 254 μm thickness 2.2 of dielectric constant and 0.0009 loss tangent^23^. The antenna was fed from the back side, as side feeds produced inconsistent results and did not guarantee robustness. We first calculated the design parameters of the patch antenna (length and width of the patch) by using well-known theoretical equations^16^, for our given substrate and desired frequency of 60 GHz. Subsequently, these parameters were optimized in CST simulations to minimize the antenna return loss (*S*_*11*_), yielding a patch with approximate dimensions of 1.50 by 1.50 mm, as briefly described in an earlier piece of abstract^24^. The final fabricated prototype is as shown in Fig. 7(a), while the calculated three-dimensional (3-D) patterns of the realized gain at 60 GHz is shown in Fig. 7(b). A maximum realized gain of 7.77 dBi is obtained at 60 GHz. The radiation pattern is symmetrical in the H-plane and fractionally asymmetrical in the E-plane. The radiation efficiency of the single element is around −0.025 dB (99%) for the operating bandwidth.

For experimental characterization, the square patch antenna was mounted on a V-band connector 380P12F5 (SigaTek) and was measured using the VNA. Figure 7(c) shows the simulated and measured S_11_. The measured resonant frequency of 60.25 GHz is very close to the simulated result of 60 GHz. The small deviation can be attributed to fabrication tolerance and soldering imperfections. The measured return loss is higher and the operating bandwidth is wider (approx. 10 GHz for S_11_ ≤ −10 dB) compared to the simulation results. The broad bandwidth can provide a reasonable frequency window to characterize the blood glucose level or a specific substance in a liquid, and can compensate for any frequency detuning from the antenna’s resonance when placed in close proximity to the skin or sample tank.

### Mechanical setup for the portable sensor

**Figure 8:**
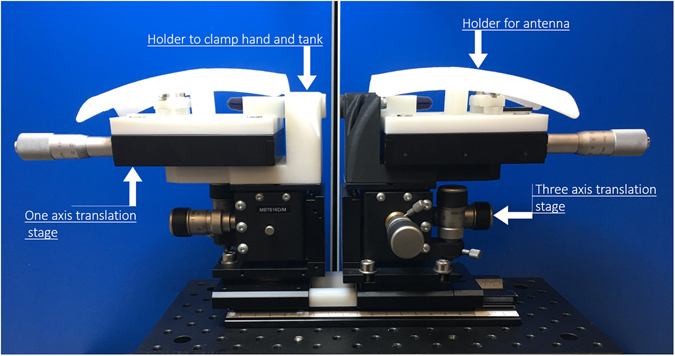
The mechanical setup. The measurement assembly containing two sets of 3-D printed holder and two sets of micro-positioner. The positioners are attached on a plate (optical table). The whole sensor can be mounted on a three axis rotational arm to align precisely against hand or ear lobe for human trial.

To realize a precision measurement system at these high frequencies, the antennas are mounted on a custom-made portable holder as shown in Fig. 8, which comprises a base, two three-axis micro positioners, and two custom 3-D printed holders. The tank is mounted on a post, and both antennas are mounted facing each other on a one-axis micro positioner with a step adjustment of 10 µm. A three-axis micro positioner equipped with a coarse and fine adjustment range of 500 μm/rev and 50 μm/rev, respectively, sits below the one-axis positioner. The custom-made 3D-printed extensions are placed on the micro positioner to avoid metal interference and prevent the antenna from any unwanted movement due to vibration. The antennas can move in all three directions independently in order to optimize the transmission coefficient *S*_*21*_ across the tank by achieving precise alignment.

### Human clinical investigation preparation and measurments

Ten healthy male subjects (n = 10, mean ± SD; age 35 ± 11 years, BMI 26.6 ± 2.5 kg/m2 and body fat 23.5 ± 7.6%) underwent an Intravenous Glucose Tolerance Test (IVGTT)^25, 26^ during the clinical investigation of the non-invasive glucose sensing system that took place at the Department of Life Sciences, University of Roehampton, London, UK. During the test, the glucose levels of the volunteers were monitored by drawing blood at regular intervals and measured it using a lab analyser (Biosen C-line)^27^. The experiments were approved by University of Roehampton ethics committee and all experiments were performed in accordance with relevant guidelines and regulations.

**Figure 9:**
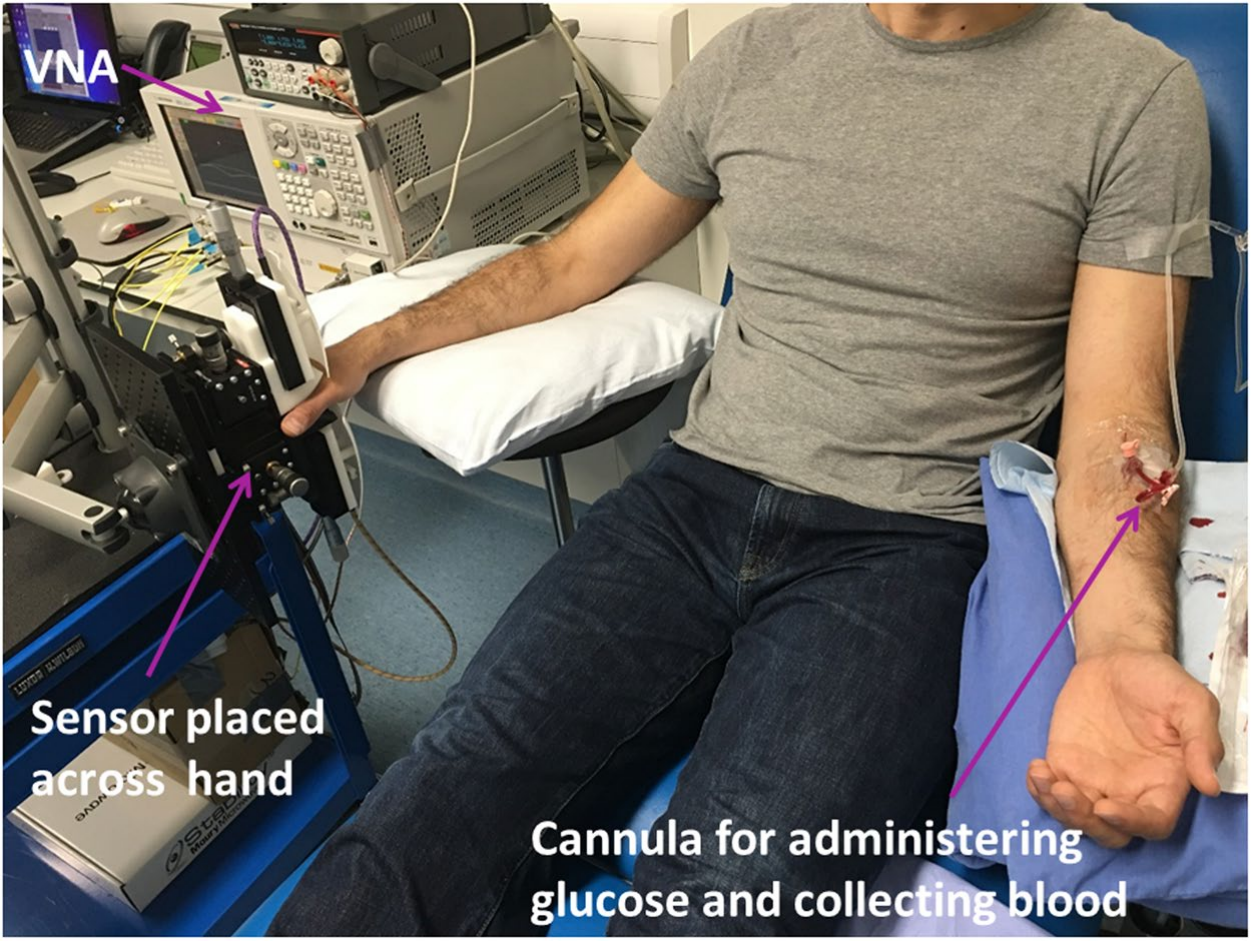
The setup for *in-vivo* glucose measurements in humans. The non-invasive system is placed across the thin tissue of right hand. Glucose was administered intravenously through the left arm. The same cannula was used to collect blood samples for measuring glucose levels during a specific amount of time.

The sensing system under investigation was placed on the right hand, in order to measure the signal variation of the thin tissue between the index and thumb fingers (Fig. 9). After placing the hand on the system, the antenna distance was adjusted so that a maximum transmission (S_21_) was obtained in the frequency spectrum where both antennas have a minimum reflection coefficient (S_11_/S_22_).

After 5 minutes baseline measurements, the sterile iv glucose load (250 mg/kg body mass; 50% glucose 0.9% NaCl saline) was administered intravenously over a period of 2 minutes via and indwelling 18″ cannula placed into a prominent contralateral antecubital vein on the left arm of each volunteer (Fig. 9). Blood was drawn at 0 and 5 minutes and at every 1 minute for the first 10 minutes after the glucose administration. After this period, the sampling frequency was every 2–5 minutes, while the non-invasive system was continuously collecting data. The test continued for approx. 60 minutes, until the glucose levels returned to normal (below 7 mmol/l) as determined by the reference instrument.

### Experiments involving human participants

Authors confirms that informed consent was obtained from all participants and University of Roehampton ethics committee approved the human clinical investigation.
